# Christmas Greeting

**Published:** 1893-12

**Authors:** 


### EDITORIAL DEPARTMENT.

F. E. DANIEL, M. D., Editor.
S. E. HUDSON, M. D., Managing Editor.
A. J. SMITH. M. D., Galveston, Associate Editor.

EDITORIAL STAFF:

PROF. J. E. THOMPSON, M. D., Texas Medical College, Galveston; Surgery.
PROF. WM. KEILLER, M. D., Texas Medical College, Galveston; Obstetrics and Gynecology.
PROF. DAVID CERNA, M. D., Texas Medical College, Galveston; Therapeutics.
PROF. A. J. SMITH, M. D., Texas Medical College, Galveston; Medicine
DR. ISADORE DYER, Tulane University; Dermatology.
DR. R. H. L. BIBB, Saltillo, Mexico; Foreign Correspondent.
DR. ROBT. MORRIS, Charity Hospital, N. O.; Clinical Reports

The JOURNAL is the official organ of the Austin District Medical Society, the West Texas District Medical Society and the Galveston County Medical Society.

### CHRISTMAS GREETING.

The TEXAS MEDICAL JOURNAL sends greeting to its thousands of readers throughout the Southland, and extends to them a sincere wish that they may, one and all, have a "Merry Christmas" and a "Happy New Year," and "many happy returns," as the bashful man unintentionally said to the bride when he sought to congratulate her.

Looking back through the vista of the years intervening since the little "Red Back" timidly made its first bow to the Texas profession, a small edition of an unpretentious little fifty-page pamphlet which seemed in a perpetual blush for its insignificant appearance, and temerity in venturing to have its "say," we recall with pride and gratitude the many words of cheer and encouragement, as well as the many acts of solid support bestowed upon the publication by the better class of Texas physicians; and to-day, at the end of nearly nine years of continued prosperity, nine-tenths of those who first rallied to its support are subscribers still, and prompt as clock-work with their annual contribution to the essential element of success—the exchequer! Thanks, again.

Yes,—through all these years their faith in the Red Back has not flickered. They have borne with our shortcomings and applauded our hits; by their steadfast devotion they have testified their faith in the ability and determination of the JOURNAL to battle for honorable, legitimate medicine, and a pure profession. Without fear or favor, it has denounced all quackery, whether *in* or *out of the profession*, and especially the masked form, and demanded that at least those who enroll themselves under the banner of organized medicine shall live up to the principles of the code, and exemplify in their lives and work the highest type of professional character; keeping steadily in view the day when our State Medical Association shall be representative of all that is purest, brightest and best in the ranks of the profession of the State; when to be a member will be,—if not "greater than a king," an honor and a distinction, of which one's children and grandchildren in their day may still be proud.

As time rings out the old year and rings in the new, we bid you God-speed; long life, honor, happiness and prosperity.

